# Truels and strategies for survival

**DOI:** 10.1038/s41598-019-45253-5

**Published:** 2019-06-20

**Authors:** Mohsen Dorraki, Andrew Allison, Derek Abbott

**Affiliations:** 1 School of Electrical & Electronic Engineering, University of Adelaide, Adelaide, South Australia 5005 Australia; 2 Centre for Biomedical Electrical Engineering (CBME), University of Adelaide, Adelaide, South Australia 5005 Australia

**Keywords:** Applied mathematics, Computational science

## Abstract

The truel is a three person competition that generalises the classic duel. In this game three players try to eliminate each other in a series of one-to-one duels until there is only one survivor. The players’ marksmanship, shooting order and strategies for choosing a target play a significant role in individual’s survival probability. Strategies such as shooting into the air (abstention), shooting at the strongest opponent, and shooting at the weakest opponent have been analysed in the previous literature. In this paper, for the first time, we consider suicidal and random strategies that can be chosen by the weaker player. We show that although there is no possible highest probability region for weakest player adopting suicidal strategy, the player may increase the survival probability via switching between suicidal and abstention strategies randomly. In addition, we demonstrate that there is a narrow survival area for the weakest player when the player aims randomly at two other opponents, and eventually the area fades away if the player fires randomly at himself or the other two opponents.

## Introduction

“Survival of the fittest” originated from Darwinian evolutionary theory as a way of describing the mechanism of natural selection^1^ stating that the fittest in the struggle for survival and reproduction increase in number, thereby leading to the design we see in nature^2^. However, in certain cases the fittest does not necessarily survive, the extinction of the dinosaurs being a classic example.

A duel consists of two gunmen, where the probability of winning relies on marksmanship and speed in unbiased conditions. A truel is a three-person expansion of a duel, in which each of the truelists can attempt to survive by shooting at opponents in order to eliminate them. Initially, the concept of a truel was introduced as a mathematical puzzle^3^, then the truel became used in the social sciences as a model of strategic interactions that can lead to seemingly paradoxical results^4–6^.

The outcome of the game is strictly dependant on the rules of the truel. In many cases truels can generate counterintuitive results and the player with the highest marksmanship does not necessarily have the greatest chance of survival. In some cases, probability-based calculations may lead to the surprising situation of “survival of the weakest”.

A wide variety of possible conditions and settings for a truel is explored in the literature, such as biasing the game by allowing the weakest player to shoot first and the best player to shoot last^7,8^. Another possibility, as a generalisation of the classic duel, is a simultaneous truel when all three players fire their bullets at the same time^9^. Moreover, winning strategies are investigated for a random truel where each player is chosen at random among the survivors in every round^10^.

The strategies that can be potentially chosen by players have been extensively investigated in previous studies. The Nash equilibrium suggests that the best action players can optimise their chances of survival via firing the bullets at the strongest remaining opponent. Surprisingly, it is shown that under certain conditions the chances of survival are improved if the weakest player fires a bullet into the air rather than targeting the strongest player^7^. The possibility of the situation in which the worst and better players are both willing to reverse the order of their moves has been investigated^11^. Previous studies investigated the concept of a truel in the case where probabilities are governed by the rules of quantum mechanics^12,13^. Other different strategies such as cooperative vs non-cooperative truels^14^ and shooting in the air by all players^15^ have also been considered.

A model of behaviour in an intense conflict situation is modelled using the theory of truels in psychological studies^16^. Some notions of equilibrium in the truels have been proposed to model political conflicts^15^. The truel has also been reinterpreted as a model for opinion spreading during debate^17^. In terms of commercial competition, it is argued that if firms or business units value their future payoffs high enough, each concentrates more on fighting the strongest opponent. Consequently, weaker companies grow stronger and the strongest grow weaker with all the parties eventually converging and remaining in the game^18^. In addition, implications for the maintenance of variation in natural populations are investigated based on *N*-player versions of the duel under constant selection^19^. The truel has also been considered to elucidate questions about war strategies, politics, marriage and reproduction^20^.

Moreover, the literature draws some interesting inferences regarding the behaviour underlying truels. Considering the eschatological views of the players in bounded and unbounded truels, it is suggested that in a finite competition players who take a more short-term or bounded view may act less responsibly, even immorally^21^. On the other hand, to the degree that the future seems to stretch out indefinitely, people are more likely take responsible strategies toward each other regarding the fact that tomorrow they may pay the price of any untoward behaviour. In the area of legal studies, the concept of truel is used as a model for exploring the issue of equality^22^, where people respond in strategic ways to legislation to maximise their interests. Therefore, the fittest party attempts to aggravate the legislature’s mistaken belief that it needs help. The party will be in the forefront of legislative activity, hiring lobbyists and buying advertisements to communicate the impression that it is the victim of disadvantageous treatment, when in reality it has the greatest chance for survival.

Previous studies have also applied the concept to a voting model showing a paradoxical result^23^. It was concluded that an initial decrease in support for a candidate later results in an upsurge in the polls for the candidate. Moreover, some studies have employed the rock-scissors-paper model to systems with three species that interact with each other and ultimately create a competitive loop^24,25^. The paradoxical effect in this model is that the least competitive species may potentially become the largest population in the ecosystem. Moreover, Parrondo’s games illustrate a paradoxical situation where losing gambling games played in a random or periodic order can result in a winning outcome^26^.

A number of suicidal strategies can be seen in a variety of species in biology. Ecological suicide is a common phenomenon in microbes, for instance, bacteria can modify the environmental pH to such a degree that it leads to a rapid extinction of the whole population^27^. In this case low density populations thrive while high density leads to ecological suicide. In addition, the number of the cells in a multicellular organism is tightly regulated by rate of cell death. In case cells are no longer required, they commit suicide using an intracellular death mechanism that is called programmed cell death or *apoptosis*^28^. Also suicidal reproduction or *semelparity* has evolved in a few mammals. Mating in the species can continue for many hours with multiple partners and after that the males all die. In fact, the males compete not by fighting, but by mating themselves to death because their sperm is in competition with the sperm of many other males. The immense pressure to produce better sperm leads to immune system failure and eventually death^29^.

In this paper, we propose some different tactics such as a suicidal strategy in the sequential truel. A sequential truel is one where three players take turns to fire one shot until the last man stands. Using the suicidal strategy the first player attempts a shot at himself whenever both the other players are alive. We also consider the case of a random strategy for the first player while other players use the strategy of targeting the strongest opponent. Note that the truel is sequential. Thus, the players take turns to shoot one bullet at a time until the last man stands.

In this paper we shall make the following assumptions:

- Players A, B, and C have a probability of hitting their chosen target of *a*, *b*, and *c* respectively, independent of their target and with $0\le a\le b\le c\le 1$.
- Each player’s marksmanship does not depend on other players.
- Each player fires sequentially in alphabetic order, and the winner is the one left standing.
- When all three players are alive, players B and C target the strongest opponent, which means B fires at C and C fires at B.

## Methods

### Targeting an opponent versus abstention

In a sequential truel, three players shoot it out and the winner is the one left standing. Respectively, player A with marksmanship *a*, player B with marksmanship *b*, and player C with marksmanship *c* fire the bullets at their chosen targets, where $0\le a\le b\le c\le 1$.

**Figure 1:**
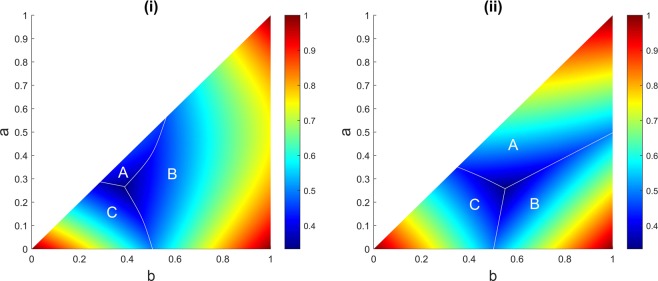
(**i**) This is the case where player A aims at the strongest opponent, and (**ii**) this is the case when play A adopts a strategy of abstention by shooting in the air during a sequential truel. Different areas (A, B, and C) show regions in the parameter space (*b*, *c*), setting $c=1$, in which each player has the highest probability of survival. The game is simulated for one million rounds for each possible combination of *a* and *b* when $0\le a\le b\le 1$ with step size of 0.001. The result is remarkable in that player A’s parameter space for survival is dramatically increased by abstention rather than firing at the strongest opponent. The colour bar represents the chance of survival.

The strategy of firing at the opponent with highest marksmanship as a target appears to be rational. In this strategy, when all three players are alive, player A fires at C, player B fires at C, and player C fires at B. Although, the naive expectation is that the strongest opponent strategy is the optimum strategy for player A, previous studies show that player A can increase his chance of survival by shooting into the air in first round or whenever both the other players are alive^7^. In particular, at any moment the sequential truel can change to a sequential duel. In some cases being the first shooter in a sequential duel is more advantageous than marksmanship, because in sequential duel the opponent’s marksmanship does not come into play until he takes his turn. In Fig. 1 we have illustrated regions in the parameter space (*a*, *b*), setting $c=1$, in which each player possesses the highest survival probability. In the simulation, the truel is repeated one million times for each combination of *a* and *b* with step size of 0.001 when *c* is forced to possess the value of 1.

Comparing Fig. 1(i,ii), it is possible for all three players to have the highest chance of survival with both strategies; however, the chance of player A’s survival dramatically increases by deciding to shoot in the air.

### Suicidal strategy

**Figure 2:**
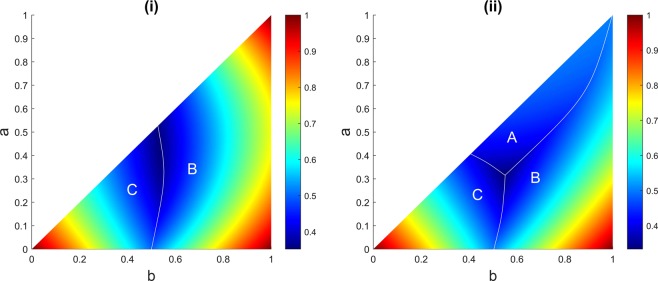
All possible survival scenarios for players A, B, and C when player A adopts a suicidal strategy, and players B and C maintain a strongest opponent strategy.

We can add an interesting twist to the truel problem by allowing player A to shoot himself, with probability *a*, whenever all other opponents are alive. The probabilities of A, B, and C surviving against their opponent using this strategy may be calculated. Players B and C still shoot at the best opponent (B aims at C, and C aims at B). Therefore, player A will not be shot at until B or C are dead, and A will be left standing in a duel with either B or C as a first shooter. All possible survival scenarios are shown in Fig. 2 for the players.

The scenarios for player A surviving with suicidal strategy illustrated in Fig. 2(i) may be considered as following:

- In first state, S_A1_, player A survives his own shot, and player B kills player C in a sequential duel. Then in the next state, S_A2_, via a sequential duel, A kills B.
- Similarly, in first state, S_A3_, player A survives his own shot, and this time player C kills player B in a sequential duel. In next the state, S_A4_, in a sequential duel A kills C.
  The probability of S_A1_ is given by Eq. 1,
  1 $${\rm{P}}({{\rm{S}}}_{{\rm{A1}}})=\bar{a}\cdot b+(\bar{a}\cdot \bar{b}\cdot \bar{c})\cdot {\rm{P}}({{\rm{S}}}_{{\rm{A1}}})=\frac{\bar{a}\cdot b}{1-\bar{a}\cdot \bar{b}\cdot \bar{c}},$$
  where $\bar{a}=1-a$, $\bar{b}=1-b$, and $\bar{c}=1-c$. The iterative nature of Eq. 1 and other following equations is due to the fact that players may kill their opponents in the first try or any other rounds. After being left standing in a duel with B or C remaining from previous step, the probability of A surviving against B is given by:
  2 $${\rm{P}}({{\rm{S}}}_{{\rm{A2}}})=a+(\bar{a}\cdot \bar{b})\cdot {\rm{P}}({{\rm{S}}}_{{\rm{A2}}})=\frac{a}{1-\bar{a}\cdot \bar{b}}.$$
  Correspondingly, the probability that C kills B conditioned on A being alive is given by
  3 $${\rm{P}}({{\rm{S}}}_{{\rm{A3}}})=\bar{a}\cdot \bar{b}\cdot c+(\bar{a}\cdot \bar{b}\cdot \bar{c})\cdot {\rm{P}}({{\rm{S}}}_{{\rm{A3}}})=\frac{\bar{a}\cdot \bar{b}\cdot c}{1-\bar{a}\cdot \bar{b}\cdot \bar{c}},$$
  and the probability of A surviving against C may calculated by
  4 $${\rm{P}}({{\rm{S}}}_{{\rm{A4}}})=a+(\bar{a}\cdot \bar{c})\cdot {\rm{P}}({{\rm{S}}}_{{\rm{A4}}})=\frac{a}{1-\bar{a}\cdot \bar{c}}.$$
  Therefore, the probability of player A surviving with suicidal strategy, *P*_*A*_, may obtained by
  5 $$\begin{array}{rcl}{{\rm{P}}}_{{\rm{A}}} & = & {\rm{P}}({{\rm{S}}}_{{\rm{A1}}})\cdot {\rm{P}}({{\rm{S}}}_{{\rm{A2}}})+{\rm{P}}({{\rm{S}}}_{{\rm{A3}}})\cdot {\rm{P}}({{\rm{S}}}_{{\rm{A4}}})\\  & = & \frac{a\cdot \bar{a}}{1-\bar{a}\cdot \bar{b}\cdot \bar{c}}(\frac{b}{1-\bar{a}\cdot \bar{b}}+\frac{\bar{a}\cdot c}{1-\bar{a}\cdot \bar{c}}).\end{array}$$
  In order to obtain the probability of player B surviving when A adopts a suicidal strategy, it is necessary to calculate the probability of the winning scenarios shown in Fig. 2(ii). The scenarios are listed here:
- In the first state, S_B1_, player A survives his own shot, and player B kills player C in a sequential duel. This state is exactly same as S_A1_. But in the next state, S_A2_, in a sequential duel, B kills A.
- In the first state, S_A3_, player A is killed by his own shot, and player B kills player C in a sequential duel.
  The probability B kills C under the condition A is killed by either of their bullets is obtained by
  6 $$\begin{array}{rcl}{\rm{P}}({{\rm{S}}}_{{\rm{B3}}}) & = & b\cdot a\\  &  & +\,\bar{b}\cdot \bar{c}\cdot b\cdot (a+a\cdot \bar{a})\\  &  & +\,\bar{b}\cdot \bar{c}\cdot \bar{b}\cdot \bar{c}\cdot b\cdot (a+a\cdot \bar{a}+a\cdot \bar{a}\cdot \bar{a})\\  &  & +\,\ldots \\  & = & a\cdot b\cdot \sum _{n=0}^{\infty }\,({(\bar{b}\cdot \bar{c})}^{n}\,\sum _{k=0}^{n}\,{(\bar{a})}^{k}).\end{array}$$
  Therefore, P_B_, the probability of player B surviving when A adopts a suicidal strategy is obtained by
  7 $$\begin{array}{rcl}{{\rm{P}}}_{{\rm{B}}} & = & {\rm{P}}({{\rm{S}}}_{{\rm{B1}}})\cdot {\rm{P}}({{\rm{S}}}_{{\rm{B2}}})+{\rm{P}}({{\rm{S}}}_{{\rm{B3}}})\\  & = & {\rm{P}}({{\rm{S}}}_{{\rm{A1}}})\cdot (1-{\rm{P}}({{\rm{S}}}_{{\rm{A2}}}))+{\rm{P}}({{\rm{S}}}_{{\rm{B3}}})\\  & = & \frac{\bar{a}\cdot b}{1-\bar{a}\cdot \bar{b}\cdot \bar{c}}(1-\frac{a}{1-\bar{a}\cdot \bar{b}})+a\cdot b\cdot \sum _{n=0}^{\infty }\,({(\bar{b}\cdot \bar{c})}^{n}\,\sum _{k=0}^{n}\,{(\bar{a})}^{k}).\end{array}$$
  Finally, the possible scenarios for player C surviving when A chooses a suicidal strategy shown in Fig. 2(ii) are listed here:
- In the first state, S_C1_, player A survives his own shot, and player C kills player B in a sequential duel. This state is exactly same as S_A3_. Then, in the next state, S_C2_, in a sequential duel, C kills A.
- In the first state, S_C3_, player A is killed by own shot, and player C kills player B in a sequential duel.

The probability that C kills B under the condition A is killed by either of their shots is given by

8 $$\begin{array}{rcl}{\rm{P}}({{\rm{S}}}_{{\rm{C3}}}) & = & a\cdot \bar{b}\cdot c\\  &  & +\,\bar{b}\cdot \bar{c}\cdot \bar{b}\cdot c\cdot (a+a\cdot \bar{a})\\  &  & +\,\bar{b}\cdot \bar{c}\cdot \bar{b}\cdot \bar{c}\cdot \bar{b}\cdot c\cdot (a+a\cdot \bar{a}+a\cdot \bar{a}\cdot \bar{a})\\  &  & +\,\ldots \\  & = & a\cdot \bar{b}\cdot c\cdot \sum _{n=0}^{\infty }\,({(\bar{b}\cdot \bar{c})}^{n}\,\sum _{k=0}^{n}\,{(\bar{a})}^{k}).\end{array}$$

Thus, P_C_, the probability of player C surviving when A chooses a suicidal strategy is given by

9 $$\begin{array}{rcl}{{\rm{P}}}_{{\rm{C}}} & = & {\rm{P}}({{\rm{S}}}_{{\rm{C1}}})\cdot {\rm{P}}({{\rm{S}}}_{{\rm{C2}}})+{\rm{P}}({{\rm{S}}}_{{\rm{C3}}})\\  & = & {\rm{P}}({{\rm{S}}}_{{\rm{A3}}})\cdot (1-{\rm{P}}({{\rm{S}}}_{{\rm{A4}}})+{\rm{P}}({{\rm{S}}}_{{\rm{C3}}})\\  & = & \frac{\bar{a}\cdot \bar{b}\cdot c}{1-\bar{a}\cdot \bar{b}\cdot \bar{c}}(1-\frac{a}{1-\bar{a}\cdot \bar{c}})+a\cdot \bar{b}\cdot c\sum _{n=0}^{\infty }\,({(\bar{b}\cdot \bar{c})}^{n}\,\sum _{k=0}^{n}\,{(\bar{a})}^{k}).\end{array}$$

**Figure 3:**
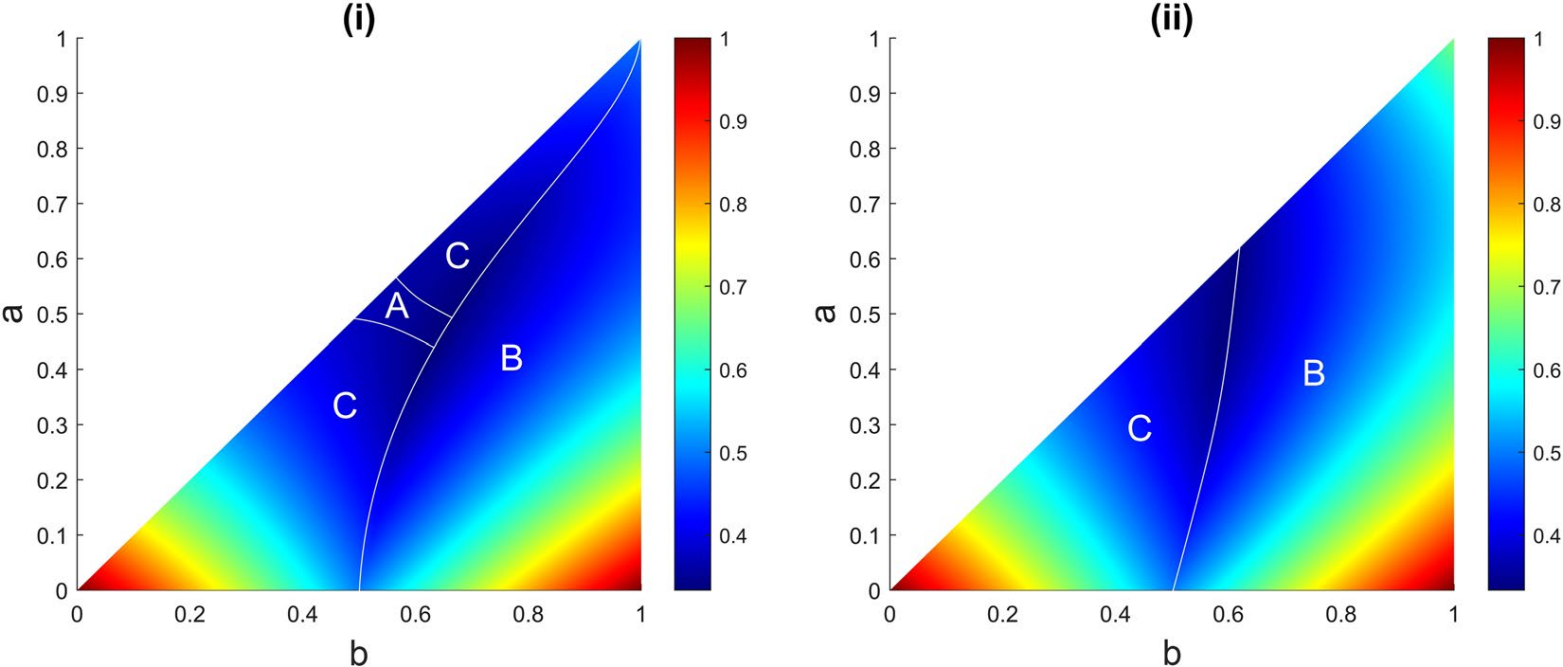
(**i**) Suicidal strategy in a sequential truel. In this case, there is no possible highest probability region for player A. (**ii**) The strategy when A randomly switches between suicidal and shooting in the air strategies. Different areas (A, B, and C) show regions in the parameter space (*b*, *c*), setting $c=1$, in which each player has the highest probability of survival. One million rounds of the game are simulated for each possible combination of *a* and *b* when $0\le a\le b\le 1$ with step size of 0.001. The colour bar represents the chance of survival.

In Fig. 3(i) we see the areas in the parameter space (*a*, *b*), setting $c=1$, in which each player possesses the highest survival probability in the suicidal strategy. As in Fig. 1, in the simulation, the truel is repeated one million times for each combination of *a* and *b* ($0\le a\le b\le 1$ with step size of 0.001).

Considering Fig. 3(i), it is not possible for player A to have the highest chance of survival in the game using a suicidal strategy. However player A can possess a large survival parameter space with a modification in the strategy. Figure 3(ii) reflects the survival regions when player A randomly switches between the two strategies: suicidal and shooting in the air.

Moreover, in case $c=1$ and with player A using a suicidal strategy, the only possible chance of survival for player A is that A must not killed in the first round and C must not survive B’s shot. This case is exactly similar to situation that player A decides to change the strategy and aim at player B when all three players are alive. Therefore, when $c=1$ both a suicidal strategy and targeting player B yield the same probability of survival for player A.

### Random strategy

Players may decide to fire the bullets randomly for different reasons such as lack of information about another opponent’s marksmanship. The distribution of winners with a random strategy in the sequential truel is investigated in previous studies^30^; however, the studies only focused on the situation that all players decide the targets randomly. We consider the scenario that player B and C continue the strongest opponent strategy, and player A uses the following random strategies: (i) decide the target randomly between B and C, and (ii) decide the target randomly among A, B, and C thus allowing the possibility of suicide.

**Figure 4:**
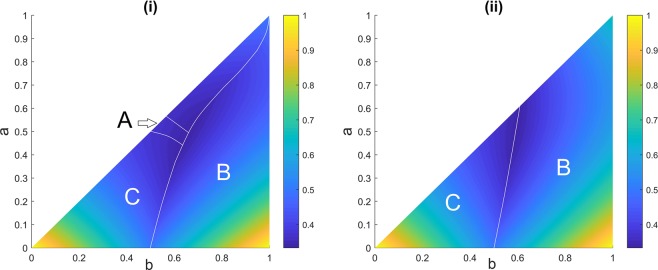
(**i**) Player A decides to randomly target opponents B or C. (**ii**) Player A randomly targets himself, B, or C. Different areas show regions in the parameter space (*b*, *c*), setting $c=1$, in which each player has the highest probability of survival. The game is simulated for one million rounds for each possible combination of *a* and *b* when $0\le a\le b\le 1$ with step size of 0.001. The colour bar represents the chance of survival.

Figure 4(i) demonstrates the areas in the parameter space (*a*, *b*), setting $c=1$, in which each player possesses the highest survival probability when A randomly chooses the target from B and C. The figure shows although the largest area corresponds to the player with intermediate marksmanship, B, it is sometimes possible for player A to have the highest chance of survival albeit over a small parameter space.

Now we may add a twist to the random strategy and combine it with the suicidal strategy as given by (ii) above. The parameter space for this strategy that A randomly fires at himself or at the other two opponents is shown in Fig. 4(ii). The narrow parameter space in Fig. 4(i) that shows when A has the highest chance of survival now totally fades away in Fig. 4(ii).

## Conclusion

It is known that under different circumstances, the fittest player in a truel does not necessarily survive. Considering different strategies in a sequential truel, it is surprising that the player with the best marksmanship does not always have the highest chance of survival.

It is already known that if the weakest player in a sequential truel begins with an abstention (e.g. by shooting his bullet in the air), he greatly increases his chance of survival. Therefore, it may seem that if he were to fire at himself (with a finite accuracy) he may still be able to survive but with a reduced parameter space—however, our results show the parameter space totally vanishes in this case. For the case when the weakest player adopts a random strategy the parameter space shrinks.

As a first step this work has considered the case where the probability of a bullet hitting its target is the same for suicide or aiming at an opponent. It would therefore be of interest for future studies to decouple this and assign different probabilities to suicidal and adversarial strategies.

Finally, we note that the literature has found the study of the truel of interest across the physical, biological, economic, and social sciences as a toy model that describes the situation where three factors conflict with each other. There are a number of important three-way conflicts (e.g. cancer cells, the immune system, and chemotherapy) that may potentially benefit from future study in this area.

We have made the Matlab code for the truels openly available on Github at https://github.com/Dorraki/truel.

The corresponding author is responsible for submitting a competing financial interests statement on behalf of all authors of the paper.
