# Publisher Correction: Truels and strategies for survival

**DOI:** 10.1038/s41598-020-62013-y

**Published:** 2020-03-17

**Authors:** Mohsen Dorraki, Andrew Allison, Derek Abbott

**Affiliations:** 1 School of Electrical & Electronic Engineering, University of Adelaide, Adelaide, South Australia 5005 Australia; 2 Centre for Biomedical Electrical Engineering (CBME), University of Adelaide, Adelaide, South Australia 5005 Australia

Correction to: *Scientific Reports* 10.1038/s41598-019-45253-5, published online 20 June 2019

The original version of this Article contained errors in the figures. The correct Figure 2 was missing, and Figures 3 and 4 were incorrectly given as Figures 2 and 3 respectively.

The figures have now been corrected in the PDF and HTML versions of the paper.

